# An update on posterior-approach blepharoptosis surgery: Influence of clinical factors on surgical outcomes

**DOI:** 10.1371/journal.pone.0343505

**Published:** 2026-02-20

**Authors:** Izabela Nowak-Gospodarowicz, Michał Kinasz, Aleksandra Kinga Kicińska, Marek Rękas

**Affiliations:** Department of Ophthalmology, Military Institute of Medicine-National Research Institute, Warsaw, Poland; PGIMER: Post Graduate Institute of Medical Education and Research, INDIA

## Abstract

**Purpose:**

To assess how preoperative clinical factors influence surgical outcomes following posterior-approach blepharoptosis repair.

**Design:**

Prospective cohort study.

**Methods:**

We conducted a prospective analysis of patients undergoing posterior-approach ptosis repair from 2022 to 2024. The primary outcome was change in marginal reflex distance 1 (ΔMRD1) at a 3-month follow-up. Secondary outcomes included inter-eye symmetry, contour, patient satisfaction (graded 0–2), surgery duration, and complication rates. We analyzed outcomes across three clinical subgroups: degree of ptosis (MRD1 ≥ 1 mm vs < 1 mm), levator function (LF > 8 mm vs 4–8 mm), and phenylephrine test result (positive vs negative).

**Results:**

A total of 231 eyes were included. Mean MRD1 improved significantly from 0.14 ± 1.5 mm preoperatively to 4.0 ± 0.8 mm postoperatively (P < 0.001). Subgroup analysis showed that patients with greater preoperative ptosis (MRD1 < 1 mm) required more extensive surgery, but clinical outcomes and patient satisfaction did not differ significantly between groups. Neither levator function nor phenylephrine test results influenced final surgical success or patient satisfaction, but preoperative ptosis severity and phenylephrine test results impacted ΔMRD1.

**Conclusions:**

Preoperative factors such as ptosis severity, levator function, and phenylephrine test result influence surgical planning but do not affect the final outcomes of posterior-approach blepharoptosis surgery. This evidence supports broader application of minimally invasive posterior approaches in clinical practice.

## Introduction

The correction of drooping eyelids (blepharoptosis) is one of the most commonly performed procedures on the eyelid [1,2]. Blepharoptosis is not only a cosmetic problem, but primarily a medical one [2]. Drooping eyelids obstruct the visual field, can modify the shape of the ocular surface inducing refractive errors, and in children can be the cause of underdeveloped vision [3]. There are many surgical methods for the correction of blepharoptosis. Clinically, they can be divided into 3 groups: methods performed on the levator muscle of the upper eyelid via the skin, methods performed from the inside of the eyelid (from the conjunctiva), and methods whose essence is the suspension of the upper eyelid on the frontalis muscle [2–4]. Methods performed from the conjunctival side, in addition to the absence of a visible skin scar, have advantages over traditional transcutaneous methods in terms of shorter surgery and recovery time, a more natural shape of the upper eyelid crease after surgery, and a shorter learning curve for the surgeon [1–7]. Disadvantages include, according to some authors, limited efficacy in the correction of minor blepharoptosis (up to 3mm with at least good levator function (LF)) [4,8,9]. There are also ongoing discussions about the algorithms used to estimate the amount of eyelid lift after surgery, since the exact mechanism of action of transconjunctival blepharoptosis surgery is not fully elucidated [4,10–12]. Clinicians wonder about the role of LF, Muller’s muscle, as well as the tests of thein preoperative administration of phenylephrine eyedrops [13–15]. Among the numerous papers on both the topic and the increasingly widely used procedures, there is a lack of prospective comparative research focusing on updating the indications for this type of procedure [12,16,17].

This study aims to update current knowledge by prospectively analyzing how severity of ptosis, levator function, and phenylephrine response influence surgical outcomes. To our knowledge, this is the first and largest prospective study on this topic.

## Materials and methods

### Study design

This was a prospective cohort study.

### Study population

The study was conducted in 2022–2024 with adherence to the rules of good medical practice and the Declaration of Helsinki. Bioethics committee approval was obtained at the Military Medical Chamber in Warsaw, Poland (No. 238/22 obtained on July 29, 2022). Study participants were recruited prospectively from August 1, 2022, to August 30, 2024. The study group included ptotic adults, whose health condition allowed participation in the study for at least 6 months. Each patient gave written informed consent for surgical treatment and participation in the study. At the same time, eligible patients had to be able to understand the requirements of the study and comply with its recommendations, as well as meet the inclusion criteria as follows: 1) men and women, of any race, over the age of 18, 2) one or both eyes with significant blepharoptosis (i.e., corneal *marginal reflex distance 1* (MRD 1)≤2 mm) or inter-eyelid asymmetry >1mm, 3) LF ≥ 4 mm, and 4) a positive or negative test result with 10% phenylephrine administered topically into the conjunctival sac. Exclusion criteria were: 1) serious illnesses or a health condition that prevented treatment and continuation in the study for a period of 6 months, 2) suspected neoplastic lesions of the orbit and eyelid or other significant skin and eyelid conditions, 3) past trauma and surgical treatment in the orbit and eyelids, 4) moderate to severe dry eye syndrome, 5) documented sensitivity to the pharmacological agents used in the study related to the preoperative, operative and postoperative procedures, 6) mental disorders or emotional instability to a degree that does not allow the participant to give informed consent to participate in the study and attend scheduled follow-up visits, 7) patient lack of cooperation or inability to perform the procedures planned in the study, 8) pregnancy and breastfeeding, or 9) lack of patient consent.

### Methods

All patients included in the study underwent customized transconjunctival surgery based on preoperative MRD1, LF, and phenylephrine test (PT), as previously described by the first author:

4mm of Muller’s m. resection to correct 1 mm of ptosis (depending on PT result: 3–12 mm) with or without upper eyelid tarsal plate resection. 1 mm of tarsus resection to correct 1 mm of residual ptosis after the PT, but leaving min. 4 mm of the upper eyelid tarsus intact [12].

Mullerectomy and tarsectomy amounts were adjusted accordingly. All procedures were performed under local anesthesia by a single surgeon (ING).

The surgical technique used was also described in the same article, in brief: the upper eyelid landmarks (the center of the pupil and the limbus of the cornea from the nose and temples) were marked on the edge of the upper eyelid. Local anesthesia with 2% Xylocaine with adrenaline (1: 100 000) was infiltrated. Traction sutures on the edge of the upper eyelid were placed. The upper eyelid was inverted on a Desmaress retractor. The determined range of the Mueller’s muscle resection and the tarsus of the upper eyelid was marked with a skin marker. Traction sutures on the Mueller muscle (e.g., Mersilk 6–0) were placed. Three absorbable mattress sutures (Vicryl 6–0) per upper eyelid tarsus were placed about 1 mm below the Putermann clamp. The determined amount of the conjunctiva and the Mueller’s muscle, as well as the appropriate amount of the upper eyelid tarsus on the edge of the Putermann clamp, was cut off with a scalpel (blade No. 11). Bleeding vessels were cauterized. Absorbable continuous sutures 8–0 were placed on the conjunctiva. A bandage contact lens was placed on the cornea. Antibiotic drops were instilled. An eyepad was placed on the eyelid [12].

The primary outcome measured was change in MRD1 (ΔMRD1) at a 3-month follow-up.

Secondary outcomes measured were: ΔMRD1 in the treated eye compared to the contralateral eye, lid contour (graded as good or not if the medial residual ptosis, lateral residual ptosis, peak or any contour changes were observed, based on before and after pictures at a 3 month follow-up), patient satisfaction on a 0–2 scale (graded anonymously as 0 = not satisfied with the results, 1 = satisfied with the results, 2 = very satisfied with results) at a 3 month follow-up, duration of surgery in minutes, and complication rate.

The primary and secondary outcome measures were compared in 3 subgroups defined by potential limiting factors:

1. Depending on the degree of preoperative blepharoptosis: patients with blepharoptosis ≤3 mm (MRD1 ≥ 1 mm) compared to patients with blepharoptosis >3mm (MRD1 < 1mm),
2. Depending on preoperative LF: patients with at least good LF (LF > 8mm) compared to patients with impaired LF (≥4 mm LF ≤ 8 mm)
3. Depending on the result of a preoperative 10%PT: patients with a negative PT result (when the MRD1 change was ≤ 1.5 mm after administration of eyedrops with 10% phenylephrine) compared to patients with positive PT (when the MRD1 change was > 1.5mm after administration of eyedrops with 10% phenylephrine)

### The statistical analysis

The data were analyzed with STATA software version 18.0 (Stata Corp LLC, College Station, TX, USA). Descriptive analysis was performed in the standard form of mean, median, mode, range, quartiles and standard deviation. For measurable features, the normality of the distribution of analyzed parameters was evaluated graphically based on a histogram. To test for normal distribution, the three customary tests (Kolmogorov-Smirnov, Shapiro-Wilk, and Anderson Darling) were performed in Stata. Standard parametric and non-parametric tests were used. The Wilcoxon pair order test was used to compare the two dependent groups. The Mann-Whitney U test was used to compare the two independent groups. For more than two independent groups, the Kruskal-Wallis test was used. The impact of selected potential limiting factors (preoperative MRD1, LF and PT, all expressed as binary variables) on ΔMRD1 was investigated with a linear regression model. The outcomes with p value < 0.05 were considered significant.

## Results

A total of 231 eyes (111 left and 120 right) were included in this study. Demographic data of the study group are presented in Table 1.

**Table 1:** Baseline characteristics of the study group.

Study population	Number of cases	Men	Women	Age
Study group	231	99	132	69.3 ± 11.4
Subgroup with MRD1 ≥ 1	86	26	60	66.7 ± 13.1
Subgroup with MRD1 < 1	145	73	72	70.8 ± 9.9
Subgroup with LF > 8mm	179	70	109	69.6 ± 11.1
Subgroup with LF ≤ 8 mm	52	29	23	68.4 ± 12.5
Subgroup with positive 10%PT (>1.5mm)	171	75	96	70.0 ± 10.3
Subgroup with negative 10%PT(≤1.5 mm)	60	24	36	67.3 ± 13.9

**Fig 1:**
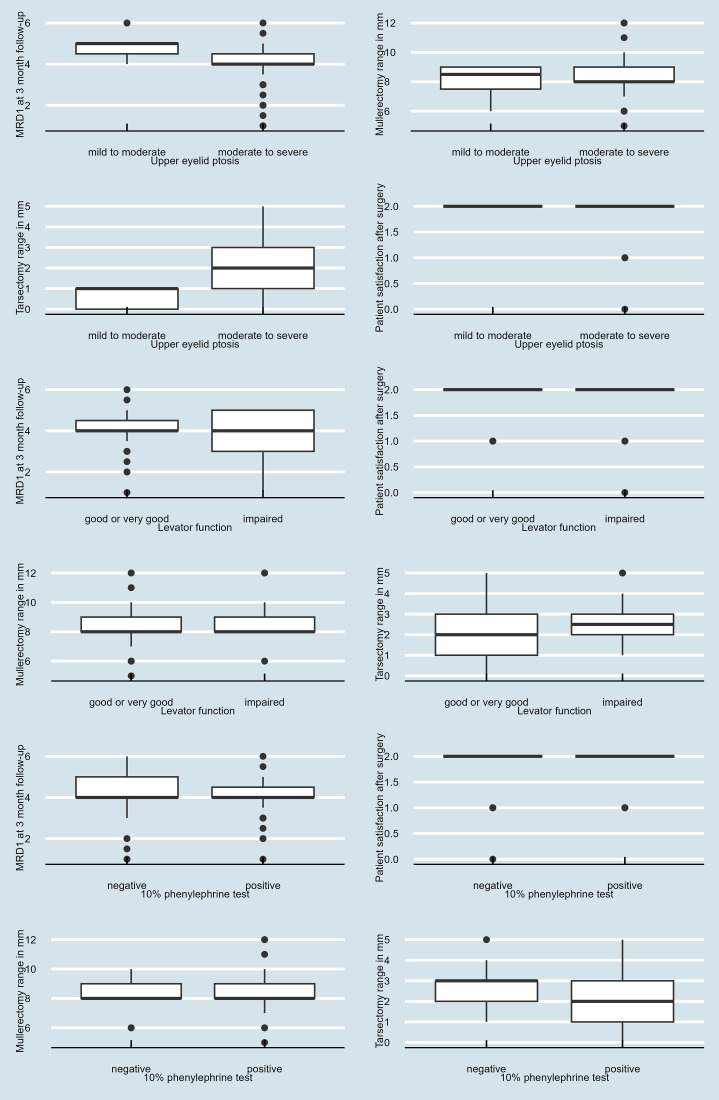
Boxplot demonstrating MRD1 changes, Mullerectomy and tarsectomy changes and patient satisfaction scores across different subgroups.

MRD1 changed significantly from 0.14 ± 1.5 mm to 4.0 ± 0.8 mm at a 3-month follow-up (Wilcoxon pair order test, V-statistics 0 with p-value <0.001). Overall patient satisfaction ranged from 1 to 2 (on a scale of 0–2). The majority of patients (98.3%) presented good eyelid contour at a 3-month follow-up. Mild residual medial ptosis was observed in 4 patients with both a high grade of ptosis and impaired preoperative LF. No patient was dissatisfied with the results of the procedure at the endpoint of the study. Inter-eyelid symmetry within 1 mm was achieved in 226 out of 228 cases (99%). Surgery time averaged 23 minutes (range 14–34). Indications for blepharoplasty were noted in 40 out of 141 patients (28.4%). Twenty-three patients (16%) reported eye discomfort despite the use of a dressing contact lens at a 2-week follow-up, which improved during further follow-ups. No significant complications were reported in the study group. Results of subgroups analyses are presented in Table 2 and Fig 1.

**Table 2:** The relationships between the compared subgroups used to compare MRD1 changes, Mullerectomy range, tarsectomy range and patient satisfaction scores.

Variable	Grouping variable	Group	N	Mean	Median	SD	IQR	P value
MRD1 at 3 month follow-up	10% phenylephrine test	negative	57	4.00	4.00	1.05	1.00	0.863
		positive	152	4.11	4.00	0.72	1.00	
	Levator function	impaired	51	3.82	4.00	1.11	1.50	0.067
		≥ good	158	4.16	4.00	0.69	1.00	
	Upper eyelid ptosis	mild to moderate	77	4.31	4.00	0.71	1.00	0.002
		moderate to severe	132	3.94	4.00	0.85	0.00	
Mullerectomy range in mm	10% phenylephrine test	negative	60	8.12	8.00	1.01	1.00	0.47
		positive	171	8.42	8.00	1.05	1.00	
	Levator function	impaired	52	8.73	8.00	1.24	1.00	0.004
		≥ good	179	8.23	8.00	0.95	1.00	
	Upper eyelid ptosis	mild to moderate	86	8.17	8.00	0.67	0.75	0.952
		moderate to severe	145	8.44	8.00	1.20	1.00	
Patient satisfaction after surgery	10% phenylephrine test	negative	60	1.80	2.00	0.44	0.00	0.143
		positive	171	1.89	2.00	0.32	0.00	
	Levator function	impaired	52	1.79	2.00	0.46	0.00	0.120
		≥ good	179	1.89	2.00	0.32	0.00	
	Upper eyelid ptosis	mild to moderate	86	1.88	2.00	0.32	0.00	0.269
		moderate to severe	145	1.86	2.00	0,37	0,00	
Tarsectomy range in mm	10% phenylephrine test	negative	54	2.52	3.00	0.99	1.00	0.001
		positive	150	2.02	2.00	1.20	2.00	
	Levator function	impaired	49	2.61	3.00	1.06	1.00	0.0005
		≥ good	155	2.01	2.00	1.16	2.00	
	Upper eyelid ptosis	mild to moderate	73	1.25	1.00	0.94	1.00	<0.0001
		moderate to severe	131	2.66	3.00	0.6	1.00	

Comparing outcome measures in subgroups, there was a statistically significant difference of ΔMRD1 in patients with preoperative MRD1 ≥ 1 mm compared to patients with preoperative MRD1 < 1mm. (Table 2, Fig 1).

The tarsectomy range differed significantly in all 3 subgroup analyses. The tarsectomy range was significantly smaller in patients with preoperative MRD1 ≥ 1 mm compared to patients with MRD1 < 1mm (P < 0.0001). In patients with LF ≤ 8 mm, there was a statistically significant difference in both the Muellerectomy range and tarsectomy range compared to patients with LF > 8mm. In patients with a positive 10% PT result there was a statistically significant difference in tarsectomy range compared to patients with a negative 10% PT result. (Table 2, Fig 1).

There was no difference in patient satisfaction, surgery duration, cosmesis or complications between subgroups (P > 0.05) (Table 2, Fig 1).

Results of linear regression modeling are presented in Table 3 and S1 File.

**Table 3:** Linear regression analysis of the impacts of potential limiting factors: preoperative MRD1, LF and PT result on ΔMRD1.

Variable	Coefficient	Robust SE	t-stat	p-value	95% CI	Significance
Const	2.4249	0.2271	10.6771	0.0	[1.9773, 2.8724]	***
Preoperative MRD1	1.847	0.1317	14.0249	0.0	[1.5875, 2.1065]	***
Levator function	−0.0664	0.2066	−0.3214	0.7482	[-0.4735, 0.3407]	
10% phenylephrine test result	0.5552	0.1506	3.686	0.0003	[0.2584, 0.8519]	***

## Discussion

Our study shows that preoperative ptosis severity, LF, and PT results influence the surgical plan but do not affect outcomes of posterior-approach blepharoptosis surgery.

It was thought that only minimal ptosis with very good LF could be corrected by a transconjunctival approach [8,9]. Current reports confirm that transconjunctival methods of ptosis correction can also be successfully used in moderate to severe ptosis, usually in procedures combining Mullerectomy and tarsectomy [5,10–12]. The aim of our study was to identify how potential limiting factors influence the outcome of posterior-approach blepharaptosis surgery. We compared outcomes in patients with minimal to moderate ptosis to those with moderate to severe ptosis. In general, we observed similar outcomes, although it should be noted that the difference in the amount of tarsectomy and Muellerectomy was significantly greater in the group of patients with moderate to severe ptosis. Another difference was noted in patients with a positive 10% PT result. The tarsectomy range was significantly smaller in this subgroup compared to patients with a negative 10% PT result (Table 2, Fig 1). Linear regression results show individuals with a positive test have a ΔMRD1 larger by 0.56 mm on average compared to individuals with a negative test (Table 3, S1 File). The only statistically significant difference in postoperative MRD1 change resulted from a comparison between the outcomes in patients with upper eyelid ptosis>3mm to patients with upper eyelid ptosis ≤3 mm (Table 2, Fig 1). Although both groups show the same median value (4.0), mild-to-moderate ptosis cases showed greater eyelid elevation than moderate-to-severe cases (4.31 vs. 3.94 mm, Table 2, Fig 1), which results from the size of the desired correction. In moderate-to-severe ptosis ΔMRD1 was significantly larger by 1.85 mm compared to the subgroup with mild to moderate ptosis (Table 3, S1 File). In patients with LF < 8mm both the Muellerectomy range and the tarsectomy range were greater than in patients with LF ≥ 8 mm (Table 2, Fig 1), but LF does not affect ΔMRD1 based on linear regression analysis (Table 3, S1 File). Most patients presented good eyelid contour at a 3-month follow-up, hovever mild residual medial ptosis was observed in 4 patients with both severe ptosis and impaired LF. This mild medial undercorrection was determinared by the anatomy of the upper eyelid and the possibility to secure and place appropriately the Putermann clamp. Thus, special care should be taken when qualifying patients with severe ptosis and impaired LF. It may be appropriate to allow for a 1:2 mm tarsectomy in these patients rather than a 1:1 ratio as suggested by some authors [18,19]. In our opinion Mueller’s anatomy and the size of the upper eyelid tarsus should be assessed before surgery in order to plan the appropiate blepharoptosis correction in this group of patients. It should be noted, however, that procedure satisfaction and cosmetic effect were not impacted by blepharoptosis size, LF and the amount of tissue resected, or results of the preoperative 10% PT. No patient was dissatisfied with the procedure outcome at the endpoint of the study (Table 2, Fig 1). Extension of the range of correction through a transconjunctival approach was achieved by combining 2 mechanisms: Mueller muscle resection and tarsectomy. It appears that additional submaximal safe tarsectomy affects the extent of blepharoptosis correction and cosmetic outcome, as described by other authors for the correction of severe blepharoptosis with poor LF via an anterior approach [20,21]. Statistically significant differences in the Mullerectomy range and tarsectomy range in subgroup comparisons may confirm this assumption (Table 2, Fig 1). Minor complications such as keratitis reported in 0.85%, undercorrection and asymmetry >1mm in 5% of cases and visible change in the contour of the upper eyelid in 1.7% of cases were noted in the previously described series of 118 eyes [12] and were to avoid with the learning curve of the surgeon. It is important to note, however, that some patients from the current study (16%) reported eye discomfort despite the use of a bandage contact lens at a 2-week follow-up, which improved during further follow-up and completely resolved at the endpoint of the study.

With the trend toward minimally invasive surgery, surgical techniques for posterior-approach ptosis repair are increasingly being turned to as quick, effective and safe procedures.

This study provides updated evidence that posterior-approach blepharoptosis surgery can achieve successful outcomes even in cases of moderate to severe ptosis and impaired LF (4–8 mm).

While greater initial ptosis and reduced LF necessitate larger tissue resections, these factors do not negatively impact final eyelid position or patient satisfaction. This challenges traditional restrictive indications and supports expanding the use of posterior-approach techniques. This may result in an increase in the popularity of transconjunctival methods in the correction of moderate to severe blepharoptosis rather than only in minimal blepharoptosis, as was standard practice in the past.

Like any study, ours also has its limitations.

One such limitation is certainly the lack of comparison stemming from blepharoptosis etiology and the lack of a comparison group treated with a different surgical technique. This is due to the fact that the study group included adult patients, mostly with involutional blepharoptosis, where there is no standard of care. We intended to check how different clinical factors, which may also be considered limiting factors, could influence final outcomes. To make our results reliable and show dependencies between subgroups, it was necessary to involve a big cohort, which we managed to achieve. Secondly, there was lack of child study participitats, what derminates the ethic’s reqiurements. Our conclusions were made based on a standard, but relatively short follow-up. Results would benefit from confirmation in a long term study. An advantage of this study is undoubtedly the large sample size, which allowed us to measure relationships in a reliable prospective manner.

## Conclusion

Preoperative ptosis severity, LF and PT results influence the surgical plan but do not affect outcomes of posterior-approach blepharoptosis surgery. These findings suggest that posterior-approach techniques can reliably be used beyond traditionally limited indications, providing a valuable contribution to the evolving practice of minimally invasive eyelid surgery.

## Supporting information

S1 Table 3. Linear regression model. (PDF)

S2 File Minimal data set. (XLSX)
