# Supplementary material for: An update on posterior-approach blepharoptosis surgery: Influence of clinical factors on surgical outcomes
Source: PLoS One. 2026 Feb 20;21(2):e0343505. doi: 10.1371/journal.pone.0343505 (PMC12922983; doi:10.1371/journal.pone.0343505)
Supplement: S1 Table — (PDF) [file pone.0343505.s001.pdf]

Table 3. OLS Regression of delta MRD1 on Binary Regressors (Robust SEs)

Variable	Coefficient	Robust SE	t-stat	p-value	95% CI	Significance
Const	2.4249	0.2271	10.6771	0.0	[1.9773, 2.8724]	***
Preop MRD1	1.847	0.1317	14.0249	0.0	[1.5875, 2.1065]	***
Levator function	-0.0664	0.2066	-0.3214	0.7482	[-0.4735, 0.3407]	
10% phenylephrine test	0.5552	0.1506	3.686	0.0003	[0.2584, 0.8519]	***

Model fit statistics

Observations: 231.0

R-squared: 0.4453

Adj. R-squared: 0.438

F-statistic (model): 60.7534

F p-value: 0.0

RMSE: 1.0558

AIC: 688.6463

BIC: 702.416

DF Model: 3.0

DF Residuals: 227.0

Diagnostic tests

Jarque-Bera stat: 17.0632

Jarque-Bera p-value: 0.0002

Skewness: 0.2643

Excess Kurtosis: 4.222

Breusch-Pagan LM stat: 16.0572

Breusch-Pagan LM p-value: 0.0011

Breusch-Pagan F stat: 5.6526

Breusch-Pagan F p-value: 0.0009

White LM stat: 18.0027

White LM p-value: 0.0062

White F stat: 3.1554

White F p-value: 0.0054

Notes

Coefficients represent the average change in delta MRD1 when the binary variable changes from 0 → 1, holding other variables constant.
Robust (HC1) SEs are reported in parentheses to correct for heteroskedasticity.
Significance levels: *** $p<0.001$, ** $p<0.01$, * $p<0.05$, · $p<0.10$.
The F-test confirms joint significance of the regressors.
Diagnostic checks show the distributional properties of residuals and possible heteroskedasticity. Inference is robust thanks to HC1 SEs.

Binary regressors legend:

1= if moderate to severe ptosis, 0=if mild to moderate ptosis

1=if positive PT, 0 if negative PT

1=if≥ 8LF, 0= if impaired LF
